# Quantitative detection and survival analysis of VBNC *Salmonella* Typhimurium in flour using droplet digital PCR and DNA-intercalating dyes

**DOI:** 10.1128/spectrum.00249-24

**Published:** 2024-07-08

**Authors:** Liyan Li, Sungwoo Bae

**Affiliations:** 1 Department of Civil and Environmental Engineering, College of Design and Engineering, National University of Singapore, Singapore, Singapore; University of Turin, Turin, Italy

**Keywords:** *Salmonella*, viability PCR, DNA-intercalating dye, DyeTox13, droplet digital PCR, food safety

## Abstract

The difficulty in detecting viable but non-culturable (VBNC) *Salmonella* by culture-dependent methods poses a risk to food safety. In our study, we applied a viability test to *Salmonella* following a lethal treatment and to flour samples inoculated with *Salmonella* to evaluate the effectiveness of viability polymerase chain reaction (PCR). Our findings revealed that the combination of both ddPCR and qPCR with those DNA-intercalating dyes could quantify viable cells at low concentrations when the plate counting method failed to detect them post-inactivation. Prolonged UV exposure did not induce cell membrane disruption, as confirmed with PMA-ddPCR, with insignificant differences in gene copies. However, samples exposed to DyeTox13 and DyeTox13 + EMA showed lower gene copy numbers, implying that enzymatic activity was decreased by UV exposure duration. In addition, temperature-dependent survival in flour revealed uniform decay rates and D values (time required for a 1 log reduction) of DNA in untreated samples across various temperatures. By contrast, different decay rates were observed with DNA-intercalating dyes (DyeTox13 and DyeTox13 + EMA), showing faster metabolic activity loss at higher temperatures in flour. The decay rates and D values, determined through plate counting and those DNA-intercalating dyes, indicated the potential presence of VBNC *Salmonella*. A strong correlation between DyeTox13 dyes and the plate counting method suggested DyeTox13 as a rapid alternative for detecting *Salmonella* in flour. The ddPCR with DNA-intercalating dyes could effectively evaluate *Salmonella* viability, facilitating more precise monitoring of VBNC in food.

**IMPORTANCE:**

*Salmonella*, a major foodborne pathogen, poses significant risks, particularly to vulnerable groups like infants, older people, and the immunocompromised. Accurate detection is vital for public health and food safety, given its potential to cause severe and life-threatening symptoms. Our study demonstrated digital polymerase chain reaction (ddPCR) with DNA-intercalating dyes for identifying the different physiological statuses of Salmonella. Also, the application of ddPCR with DNA-intercalating dyes offers quantification of viable cells post-disinfection as an alternative method in food. Utilizing ddPCR and DNA-intercalating dyes, we enhanced the detection of VBNC *Salmonella*, a form often undetectable by conventional methods. This innovative approach could significantly improve the precision and efficiency of detection for viable *Salmonella*. By providing deeper insights into its transmission potential, our method is a critical tool in preventing outbreaks and ensuring the safety of food products. This research contributes substantially to global efforts in controlling foodborne illnesses and safeguarding public health.

## INTRODUCTION

*Salmonella*, a notable bacterial pathogen, poses a significant risk to food safety due to its potential to cause fever, diarrhea, gastroenteritis, and sepsis in humans. In 2023, a *Salmonella* outbreak linked to flour consumption resulted in 14 infections across 13 U.S. states, as reported by the U.S. Food and Drug Administration and the Centers for Disease Control and Prevention (1). Furthermore, between 2017 and 2022, raw flour was implicated in 22 recalls or outbreaks due to contamination with *Salmonella* or *E. coli* O157:H7 (2). These incidents challenge the common misconception that flour’s dry nature renders it a low-risk ingredient, as bacterial pathogens such as *Salmonella* and *E. coli* O157:H7 can persist in wheat flour for prolonged periods (3).

The detection of pathogens in flour is complicated, particularly when they enter the viable but non-culturable (VBNC) state. In this state, bacteria remain metabolically active but undetectable by conventional plating methods, posing significant public health and food safety risks (4–8). It was known that these bacteria could maintain their pathogenicity in the VBNC state (9), highlighting the critical need for accurate and rapid detection methods for microbiological monitoring and ensuring food safety (9, 10).

Pathogen detection typically involves culture-based methods, microscopic examination, and molecular techniques, each with distinct advantages and limitations. Molecular techniques, especially, have gained prominence as rapid and reliable alternatives for pathogen detection in water and food (11). Particularly, viability polymerase chain reaction (PCR), the PCR with DNA-intercalating dyes, such as propidium monoazide (PMA) and ethidium monoazide (EMA), were used to assess cell viability in food and water (12–17). The DNA-intercalating dyes’ strategy to determine cell viability was based on their ability to penetrate the compromised membranes of dead or damaged cells (18, 19). Once photoactivated, these dyes formed a covalent bond with DNA, thereby preventing the PCR amplification of DNA from dead cells. Recent studies utilized new DNA-intercalating dyes such as DyeTox13 and DyeTox13+ EMA in conjunction with quantitative PCR, providing valuable insights into the bacterial physiological status (8, 20, 21).

However, quantitative PCR (qPCR) was known for limitations such as sensitivity at low target concentrations, effects of PCR inhibitors, and sample matrix effects on PCR amplification efficiency (22–24). However, digital PCR (dPCR) offers advantages over the qPCR technique because it provides absolute quantification, higher precision and sensitivity, and reduced effects of PCR inhibitors (25, 26). Furthermore, dPCR was less susceptible to variations in amplification efficiency and sample matrix effects on PCR amplification (27, 28), resulting in more sensitivity than qPCR in counting low-copy number cells (29, 30).

Droplet digital PCR (ddPCR) is increasingly used to detect pathogenic bacteria in clinical and environmental assessments (31, 32). By contrast, recent studies have explored the combination of ddPCR with PMA to quantify viable pathogens like *Campylobacter* and *E. coli* O157:H7 (33–35). Understanding how *Salmonella* survives and persists after inactivation, especially in flour, is a significant research gap. In this study, our objectives were (i) to evaluate the application of ddPCR in conjunction with DNA-interacting dyes (PMA, DyeTox13, and DyeTox13+ EMA) for assessing *Salmonella* cell viability (ii), to assess the efficacy of inactivation methods using ddPCR with DNA-intercalating dyes, and (iii) to determine the persistence and viability of *Salmonella* in wheat flour. By implementing viability ddPCR assays, our research seeks to elucidate *Salmonella* cells’ viability state following inactivation and evaluate their survivability in wheat flour.

## MATERIALS AND METHODS

### Bacteria strains and culture condition

*Salmonella* Typhimurium ATCC 14028 (*S*. Typhimurium) served as a representative model for foodborne pathogens. An isolated colony of *S*. Typhimurium from an overnight tryptic soy agar (TSA, DifcoTM, USA) was inoculated into 100 mL of sterile tryptic soy broth (TSB, Sigma-Aldrich, USA) and incubated for 12 hours at 37°C with a shaking speed of 200 rpm. Subsequently, the bacterial cells were harvested, washed, and resuspended in 50 mL of 1 × phosphate-buffered saline (1 × PBS). The final concentration of *S*. Typhimurium was adjusted to OD_600_ equal to 0.25 (approximately 10^8^ CFU/mL) before subsequent treatments.

### Treatment of pure-cultured *Salmonella*

For pasteurization, 500 µL suspensions of *S*. Typhimurium suspensions in 1 × PBS were subjected to a temperature of 63℃ for 30 minutes using a standard laboratory heat block (Bio Laboratories, Singapore). Subsequently, samples were cooled on ice before the dye treatments. UV inactivation was performed on 5 mL of bacterial suspensions spread evenly in 30 mm Petri dishes. These Petri dishes were positioned in a Type A2 biological safety cabinet (BSC) equipped with a timed UV system (Thermo Fisher Scientific, USA). The UV intensity was consistently set at 0.055 mW/cm^2^ as verified by a standard laboratory radiometer (IL 1400A Radiometer, International Light). Exposure durations of 10, 20, and 30 minutes were employed, corresponding to 33, 66, and 99 mJ/cm^2^, respectively. As a control, cells in 1 × PBS buffer without UV treatment were also evaluated in subsequent dye assays. For both pasteurization and UV-treated cells, the loss of cell cultivability was determined in triplicate by plate counting on TSA.

### Dye-exposure (PMA, DyeTox13, DyeTox13+EMA)

Propidium monoazide (PMA, Biotium, USA), DyeTox13 Green C-2 Azide (DyeTox13, Setareh Biotech, USA), and ethidium monoazide bromide (EMA, Biotium, USA) were prepared as 20 mM stock solution in 20% dimethyl sulfoxide (DMSO, Sigma-Aldrich, Singapore) and stored at −20°C, shielded from light. For treatments, 500 µL of cell suspension was exposed to either PMA or DyeTox13 to achieve a final concentration of 50 µM. A combined treatment was also prepared, introducing both DyeTox13 and EMA, with the final concentration of 50 µM and 25 µM, respectively. Following dye addition, the suspensions were thoroughly mixed *via* vertexing and incubated in the dark at ambient temperature for 10 minutes. The samples were then illuminated for 15 minutes using a PMA-Lite LED Photolysis device (Biotium, USA). Post-illumination, cells were centrifuged at 5,000 × *g* for 10 minutes to pellet them for subsequent DNA extraction.

### Fluorescence microscopy

For fluorescence microscopy involving CMFDA/PI staining, solutions of CMFDA (Thermo Fisher Scientific, USA) and propidium iodide (PI, Thermo Fisher Scientific, USA) were allowed to equilibrate at room temperature for 30 minutes in darkness. The bacterial suspensions (1 mL) post-pasteurization and UV exposure were transferred into microcentrifuge tubes and centrifugated at 5,000 × *g* for 5 minutes. The supernatant was discarded, and the pellet was resuspended in 1 × PBS. The bacterial suspension was then treated with CMFDA to a final concentration of 5 µM and incubated at 35°C for 5 minutes. PI was added to achieve a final concentration of 1.4 µM, followed by a 5-minute incubation at room temperature. The samples were centrifuged again at 5,000 × *g* for 5 minutes, and then the pellet was resuspended in 1 mL of 4% Paraformaldehyde (PFA, Sigma-Aldrich, USA) for cell fixation. After a 10-minute fixation period, the cells were centrifuged again and resuspended in 1 × PBS. Finally, the bacterial samples were analyzed under a fluorescence microscope (Nikon, ECLIPSE 90i).

### DNA extraction, primer design, and construction of plasmid DNA

Genomic DNA (gDNA) was isolated from both the dye-treated and untreated 500 µL samples using a GeneJET Genomic DNA Purification Kit (Thermo Fisher Scientific, USA), following the manufacturer’s instructions. The concentrations of DNA were determined using a NanoDrop 2000 spectrophotometer (Thermo Fisher Scientific, USA).

The primer sequences used for quantitative PCR (qPCR) and droplet digital PCR (ddPCR) were as follows: forward primer 5′- GGCGATATTGGTGTTTATGGGG-3′ and reverse primer 5′-CCGTGGTCCAGTTTATCGTT-3′. The amplicon size is 244 bp, with a melting temperature (T_m_) of 60°C.

The target gene, *invA*, was inserted into a plasmid pGEM-T Easy Vector (System II, Promega) and cloned into JM109 competent cells. A plasmid containing the PCR amplicon was extracted from a recombinant cell using a QIAprep Spin Miniprep Kit (Qiagen, Valencia, CA, USA). The plasmid DNA (pDNA) concentration was quantified using the Qubit double-stranded DNA (dsDNA) High Sensitivity (HS) Assay Kit (Invitrogen, USA).

### qPCR and ddPCR

qPCR assays were executed using the ABI Step-One-Plus Real-Time PCR system (Applied Biosystems, USA) to amplify gDNA, with reactions in a MicroAmp optical 96-well reaction plate. Each 20 µL qPCR, conducted in triplicate, comprised 1 µL of gDNA, 10 µL of 2× PowerUp SYBR Green PCR Master Mix (Applied Biosystems, USA), and 1 µL each of 10 µM forward and reverse primers. The qPCR cycling conditions were as follows: initial denaturation at 95°C for 10 minutes, then 40 cycles of 95°C for 15 seconds, and annealing at 60°C for 60 seconds. A melt curve analysis was conducted for all SYBR Green real-time PCR assays to verify the specificity of the primer.

For ddPCR, all consumables and equipment were provided by Bio-Rad (California, USA), including the data analyzing software. Each of the 20 µL ddPCRs contained 1 μL of gDNA, 10 μL of 2 × QX200 ddPCR EvaGreen Supermix, and 1 μL of 10 µM forward and reverse primers; the rest amount of the ddPCR was filled with PCR-grade sterile water (HyClone, USA). 70 μL of QX200 Droplet Generation Oil for EvaGreen and prepared 20 μL ddPCR reagent were added to DG8 Cartridge while covered by DG8 Gasket before being placed into QX200 Droplet Generator. After the droplets generation step in the cartridge, droplets were transferred into ddPCR 96-well plates and sealed by PCR plate heat seal foil in the PX1 PCR Plate Sealer, followed by conventional thermal cycling protocol: 95°C for 10 minutes, followed by 40 cycles of 95°C for 15 seconds and 60°C for 60 seconds, and then 98°C for 10 minutes with the ramp speed was 2.5 °C/s during the whole process. Finally, the 96-well plate was loaded into the QX200 Droplet Reader, and the data will be analyzed using QuantaSoft Version 1.7.4.

### Standard curve, LOD, and LOQ analysis

Standard curves were generated for both qPCR and ddPCR. A 10-fold serial dilution pDNA containing the *invA* target gene was used to test the analytical sensitivity, linearity, and dynamic range of ddPCR and qPCR assays. The concentration of pDNA used to prepare standard DNA serial dilution was determined with a NanoDrop 2000 Spectrophotometer (Thermo Fisher Scientific, USA). To assess samples with low-gene-copy numbers, we determined the limit of detection (LOD) and limit of quantification (LOQ) using the same pDNA. The dilutions ranged from 10^0^ to 10^7^ and 10^0^ to 10^5^ copies/reaction in replicates for qPCR and ddPCR, respectively. LOD and LOQ calculation in qPCR were analyzed by the Ct values against each dilution level (Log10 form), calculated by the equations: LOD = (3.3 × standard deviation of lineal regression)/slope of the regression line, and LOQ = LOD × (10/3), following the conversion to the absolute gene copies for analysis. For ddPCR, the LOD was defined as the lowest concentration with a confidence level (often 95%) where positive droplets can be reliably distinguished from background noise (negative droplets). As LOQ calculation involves the determination of the concentration at which a certain number of positive droplets is significantly higher than the background signal, in this study, LOQ is set at a concentration one dilution level above the LOD where accurate and precise quantification is applied (21, 24, 26, 36).

### *Salmonella* Typhimurium persistence in flour across varying temperature

Following the bacterial incubation described in Section “Bacteria strains and culture condition”, *S*. Typhimurium was inoculated into flour at varying concentrations. The final concentrations of *S*. Typhimurium were adjusted to the concentration of approximately 4, 5, 6, and 7 Log10 CFU/mL for the flour inoculation. All-purpose plain flour was purchased from a local supermarket in Singapore. The background microflora of the flour was determined through three random measurements of 1 g samples. For each concentration (4, 5, 6, and 7 Log10 CFU/mL), 10 mL of *S*. Typhimurium suspension was pelleted and incorporated into 10 g plain flour in triplicates using the sterile stomacher bags to ensure homogeneity. These inoculated 10 g flour samples were then used to further inoculate 90 g samples, resulting in final *Salmonella* concentrations of 3, 4, 5, and 6 Log10 CFU/g. The mixture was agitated in a laboratory incubator (Palico Biotech Pte Ltd, SG) at 200 rpm for 10 minutes. The flour samples were then stored under three different distinct temperatures: 4°C, room temperature (25°C), and 37°C. An additional 100 g of flour samples without *Salmonella* served as a control. To mitigate the impact of a_w_ on the storage conditions of *S*. Typhimurium in flour, we placed inoculated samples in various temperature environments. These environmental conditions were monitored using a humidity meter (DWEII, SG) to achieve a target water activity of a_w_ = 0.45 ± 0.05. To verify the uniform distribution of the inoculum, we randomly selected 1 g of *Salmonella*-inoculated flour samples (3, 4, 5, and 6 Log10 CFU/g) and performed enumeration on TSA plates.

The experimentation involved incubating the flour samples for 3 weeks, with sample collection points at 0, 3, 7, 14, and 21 days, conducted under three temperatures (4°C, 25°C, and 37°C). Subsequently, 1 g of flour samples was randomly chosen for enumeration on TSA plates for the cultivability test at each collection time point. Meanwhile, 1 g of flour samples was transferred to 10 mL of 1 × PBS buffer as a final concentration of 2, 3, 4, and 5 Log10 CFU/mL. Then, four 500 μl aliquots from each flour sample were taken for dye exposure, referring to Section “Dye-exposure (PMA, DyeTox13, DyeTox13+EMA),” followed by DNA extraction and PCR-related tests (qPCR and ddPCR).

### Plate counting and FDA experiment

Flour samples inoculated with *Salmonella* were taken at the incubation time points of 0, 3, 7, 14, and 21 days for cultivability tests. From each temperature condition (4°C, room temperature, and 37°C), 1 g samples with varying *Salmonella* concentrations were suspended in 10 mL of 1 × PBS buffer. After brief agitation, the samples were centrifuged at 500 *g* for 5 minutes, and the supernatant was reserved for subsequent fluorescein diacetate (FDA) assays. Enumeration was conducted on TSA agar plates, with each condition tested in triplicate.

To stain *Salmonella*-inoculated flour samples with FDA, we dissolved FDA (Thermo Fisher Scientific, USA) in acetone to obtain a 2 mg/mL stock solution, stored at −20°C before usage. The *S*. Typhimurium suspension was adjusted to OD_600_ equal to 0.25 in 1 × PBS before the 10-fold serial dilution for standard curve generation. After the *Salmonella*-inoculated flour samples were suspended and centrifuged in 1 × PBS, FDA was added to aliquots of bacteria culture/flour samples to a final 100 µg/mL concentration. The samples were incubated on a rotary shaker (150 rpm) at 30°C for 60 min. After incubation, acetone (50% vol/vol) was added to terminate the FDA hydrolysis. Cell debris was eliminated by centrifugation at 5,000 × *g* for 5 minutes, and the supernatants were then filtered. The fluorescein concentration was measured at 490 nm using a spectrophotometer (Hitachi U-2800 UV–Vis Spectrophotometer).

### Dye-exposure experiment for flour samples

After *Salmonella*-inoculated flour samples were obtained as described above in the section “Salmonella Typhimurium persistence in flour across varying temperature,” each 500 µL aliquot of flour sample suspension was taken and treated as in the section “Dye-exposure (PMA, DyeTox13, DyeTox13+EMA)” before the DNA extraction procedure. Subsequently, gDNA obtained from flour samples after dye treatment was quantified by qPCR and ddPCR for data analysis.

### Data analysis

The effects of PMA, DyeTox13, and DyeTox13 + EMA on both treated and untreated samples were assessed using ΔCt values in the qPCR analysis. These values represent the difference in results under various dye exposures and are calculated by subtracting the Ct value of untreated samples from that of dye-treated samples. The error bars in this study’s figures represent the standard deviations derived from three biological replicates. For ddPCR, data processing was performed using QuantaSoft Version 1.7.4. The distribution ratio of positive droplets to the total droplets was evaluated using Excel and Prism 8.0. Amplitude figures were directly extracted from the QuantaSoft software.

## RESULTS

### The sensitivity and precision of ddPCR for *S*. Typhimurium quantification

**Fig 1:**
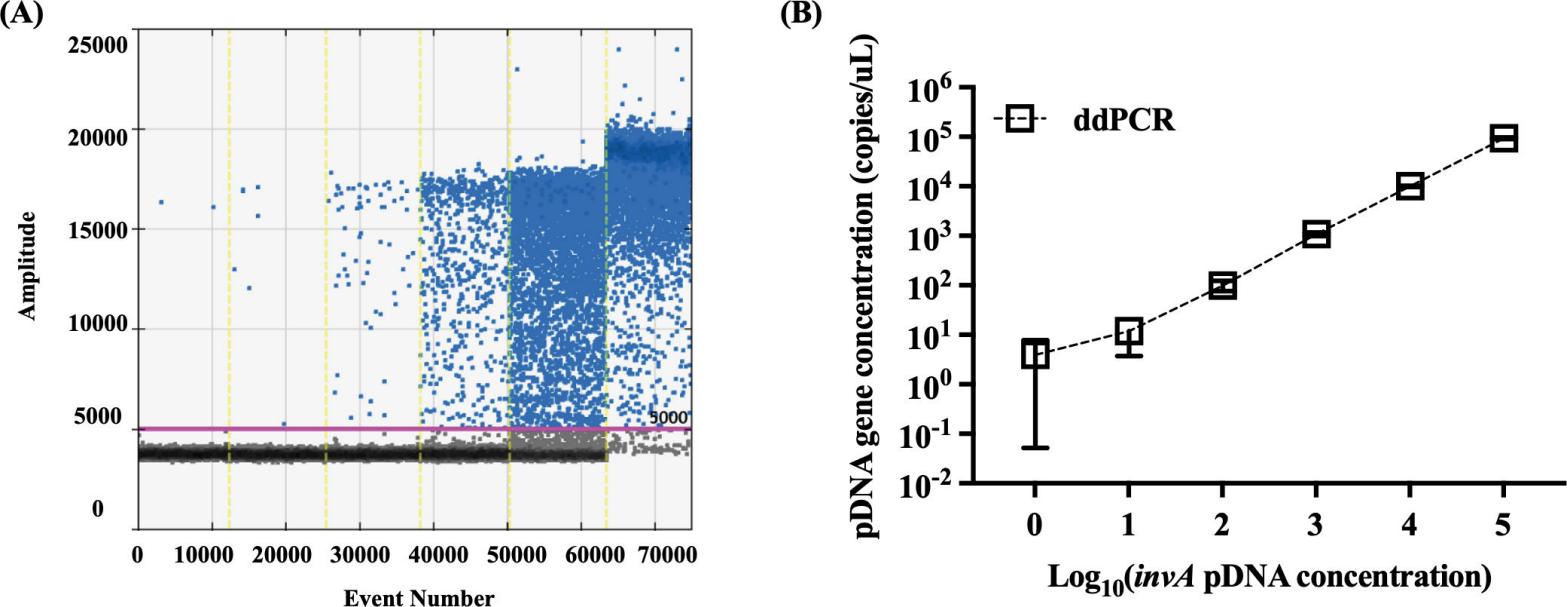
The validation of ddPCR for quantification of *S*. Typhimurium’s *invA* gene under 10-fold serial-diluted pDNA. (**A**) ddPCR amplitude results of positive droplet distribution; (**B**) Standard curve of ddPCR assay for detecting *S*. Typhimurium’s *invA* gene.

In this study, the ddPCR was utilized to detect the *invA* gene of *S*. Typhimurium in a series of 10-fold serial dilutions of pDNA, ranging from 10^5^ to 10° copies per reaction (Fig. 1). The ddPCR assay consistently and quantitatively detected the *invA* gene across all dilution levels, highlighting its precision. A linear relationship was observed between the logarithm of the initial pDNA copy number and the number of droplets testing positive for the *invA* gene, yielding a coefficient of determination (R²) greater than 0.99. This indicated a high degree of linearity in the assay. Furthermore, the sensitivity and precision of ddPCR were evaluated using *Salmonella invA* pDNA. For instance, in Table 1, at the lowest concentration, the Poisson confidence level ranged from 0.50 to 1.02, suggesting that the measured concentrations of the target molecule (*invA*) closely approximated the expected value.

**TABLE 1:** The sensitivity and precision of ddPCR assay in *S*. Typhimurium’s *invA* gene under 10-fold serial-diluted pDNA

Expected *invA* concentration in plasmid (copies/μL)	ddPCR		
	Mean (gene copies/μL)	PoissonConfMax (gene copies/μL)	PoissonConfMin (gene copies/μL)
1 × 10^0^	3.17 × 10^0^	1.02 × 10^0^	0.50 × 10^0^
1 × 10^1^	1.05 × 10^1^	2.05 × 10^1^	4.50 × 10^0^
1 × 10^2^	9.84 × 10^1^	1.24 × 10^2^	7.67 × 10^1^
1 × 10^3^	1.08 × 10^3^	1.16 × 10^3^	9.94 × 10^2^
1 × 10^4^	1.00 × 10^4^	1.03 × 10^4^	9.74 × 10^3^
1 × 10^5^	9.40 × 10^4^	9.80 × 10^4^	9.02 × 10^4^

A comparison between ddPCR and qPCR revealed a significant difference in the LOD and LOQ. Presence/absence analysis demonstrated that ddPCR yielded higher positive detections, emphasizing its effectiveness in identifying the target gene. The LOD and LOQ are critical quantitatively, defining the lowest concentration at which a target is reliably detectable and quantifiable. In this context, qPCR exhibited a LOD of 8 copies/µL and a LOQ of 550 copies/µL for the *invA* gene, corresponding to Ct values of 36 and 30, respectively. By contrast, ddPCR, with its ability to detect absolute gene numbers, achieved a lower LOD of 3 copies/µL (Table 1). The conservatively estimated LOQs for ddPCR were approximately at 3 × 10^1^ copies/µL. Therefore, the lower LOD and LOQ of ddPCR underscored its superior sensitivity in detecting low-abundance targets, attributed to digital partitioning of reactions and absolute quantification.

### Evaluating cell viability in *S*. Typhimurium post-pasteurization using DNA-intercalating dyes with ddPCR and qPCR techniques

This study systematically evaluated the effectiveness of DNA-intercalating dyes in conjunction with ddPCR and qPCR for differentiating viable from non-viable *S*. Typhimurium cells post-pasteurization. We utilized three distinct DNA intercalating dyes: PMA for membrane-intact cells, DyeTox13 for metabolically active cells associated with both intracellular and extracellular enzymes, and DyeTox13 + EMA for metabolically active cells from only intracellular enzymes. This approach aimed to determine the precision and sensitivity of ddPCR compared to qPCR.

**Fig 2:**
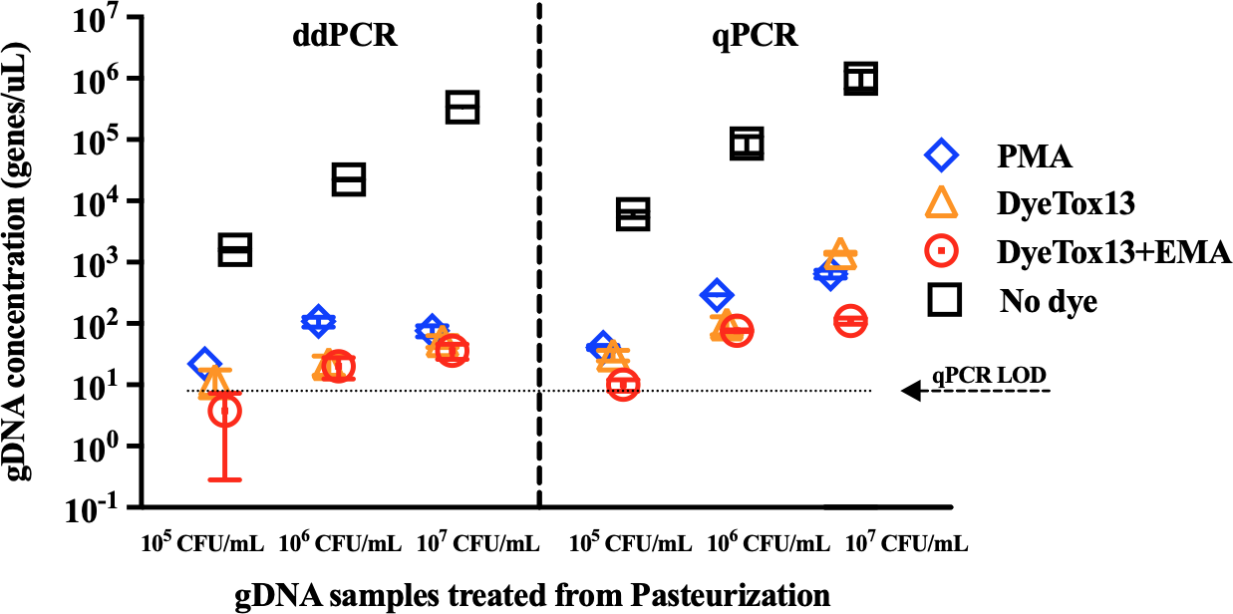
Quantification of viable cells using PMA-, DyeTox13-, DyeTox13 + EMA dye treatment, and no dye treatment when *S*. Typhimurium was treated by pasteurization. The genomic DNA extracted after pasteurization under 10^7^, 10^6^, and 10^5^ CFU/mL *S*. Typhimurium cell concentrations were measured using qPCR and ddPCR assays for comparison. qPCR LOD dot line indicated the samples’ concentration was below the detection limit in the qPCR assay.

Our experimental design involved subjecting *S*. Typhimurium to pasteurization at three initial cell concentrations (10^7^, 10^6^, and 10^5^ CFU/mL). Plate count assays post-pasteurization indicated no culturable *S*. Typhimurium cells. Nonetheless, both qPCR and ddPCR analyses detected the presence of *S*. Typhimurium in all samples (Fig. 2). A significant observation was the disparity in gDNA between samples treated with the DNA-intercalating dyes and untreated samples, suggesting that DNA-intercalating dyes with both PCRs could effectively discriminate viable from dead cells (Fig. 2). For example, at a concentration of 10^6^ CFU/mL, the *invA* gene concentrations were 1.08 × 10^2^, 2.01 × 10^1^, 3.80 × 10^0^, and 2.24 × 10^4^ gene copies/μL for samples treated with PMA, DyeTox13, DyeTox13 + EMA, and untreated sample, respectively (Table S1).

Our findings have significant implications for the field of food microbiology. DNA-intercalating dyes used with ddPCR and qPCR significantly inhibited DNA amplification in post-pasteurized *Salmonella* across various concentrations (Table S1). A comparative analysis between ddPCR and qPCR showed that ddPCR consistently yielded lower mean gene copies in samples treated with DNA intercalating dye. Also, these results highlighted the dyes’ capability to differentiate between bacterial states post-pasteurization, particularly identifying cells transitioning to a membrane-compromised state. This finding revealed that pasteurization induced cell membrane disruption, as PMA indicated (membrane integrity). Also, both ddPCR and qPCR suggested that a fraction of *S*. Typhimurium might transition into a VBNC state post-pasteurization, retaining enzymatic activity (Table S1). This observation, derived from ddPCR and qPCR analyses, highlighted the complexity of bacterial survival post-pasteurization (e.g., the transition into VBNC) and the potential implications for food safety and microbial assessment.

### The application of DNA-intercalating dyes for assessing cell viability post-UV-exposure

**Fig 3:**
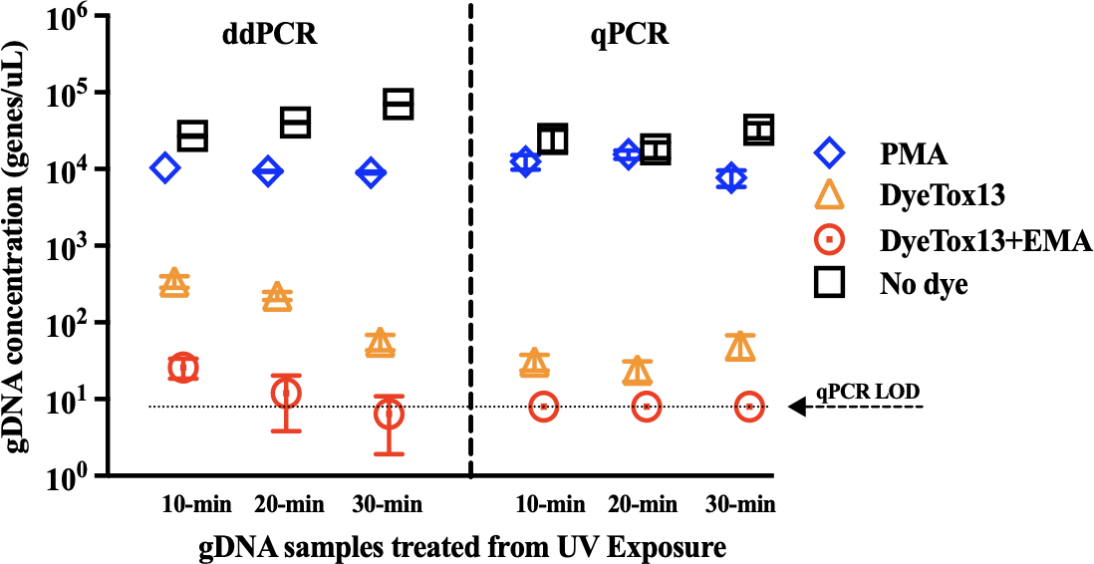
Quantification of viable cells using PMA-, DyeTox13-, DyeTox13 + EMA dye treatment, and no dye treatment when *S*. Typhimurium was inactivated by UV exposure. The genomic DNA extracted after UV inactivation under 10-, 20-, and 30-minute exposure durations were measured using qPCR and ddPCR assays for comparison. qPCR LOD dot line indicated the samples’ concentration was below the detection limit in the qPCR assay.

This study investigated the efficacy of UV inactivation on *S*. Typhimurium, focusing on the bacterium’s differential responses to various UV dosages and the application of DNA-intercalating dyes in ddPCR and PCR techniques. Figure 3 illustrates significant differences in *S*. Typhimurium concentrations when treated with DyeTox13 and DyeTox13 + EMA. Samples exposed to DyeTox13 and DyeTox13 + EMA showed lower gene copy numbers, ranging from 10^1^ to 10^2^ copies/μL (Table S2). By contrast, untreated DNA and PMA-treated samples displayed gene copy numbers from 10^4^ to 10^5^ copies/μL in both qPCR and ddPCR assays (Table S2). Despite varying UV intensities, untreated samples and those treated with PMA consistently displayed high gene copy numbers with statistically insignificant differences (*P*-value > 0.05). Notably, prolonged UV exposure did not induce cell membrane disruption, as indicated by the non-significant difference in UV exposure (*P*-value > 0.05). Table S2 showed that qPCR failed to detect samples exposed to DyeTox13 + EMA, falling below the LOD. Conversely, ddPCR successfully detected these samples, even at low concentrations ranging from 6.4 × 10^0^ to 3.4 × 10^2^ copies/μL, highlighting ddPCR’s precision and sensitivity in detecting lower gene copy numbers and metabolically active cells post-UV inactivation.

The integration of EMA with DyeTox13 enhanced the assay’s ability to assess metabolic activity in UV-treated cells by reducing PCR signals from UV-inactivated cells, thereby diminishing the detection of extracellular enzymatic activity. The ddPCR analyses using DyeTox13 and DyeTox13 + EMA indicated that most *S*. Typhimurium cells subjected to UV radiation ceased their metabolic activities. Furthermore, the results revealed a persistent presence of cells with intact membranes even after prolonged UV exposure. This implied that the impact of UV treatment on *S*. Typhimurium could be related to enzymatic activity or cellular metabolism rather than to direct damage to membrane permeability due to low UV intensities.

### *S*. Typhimurium viability test using CMFDA/PI cell counting

To evaluate the effectiveness of viability PCR using PMA and DyeTox13, we employed CMFDA and PI to assess the viability of *S*. Typhimurium cells following inactivation, including pasteurization and UV treatment, as shown in Fig. S1 and S2. Fluorescence microscopy showed that viable cells emitted green fluorescence after CMFDA staining, while cells with compromised membranes exhibited red fluorescence upon PI staining. CMFDA staining indicated that a small fraction (less than 6%, approximately 2.13 × 10^1^ cells count/μL) of bacteria, initially at a concentration of 10^5^ CFU/mL, retained enzymatic activity post-pasteurization (Table 2). Notably, plate counting assays did not detect any bacteria (data not shown), suggesting that a subset of *Salmonella* cells might maintain enzymatic activities post-pasteurization. In addition, PI staining post-pasteurization showed that most bacterial membranes were compromised, with cell counts ranging from 1.28 × 10^2^ to 9.55 × 10^3^ cells/µL (Table 2), supporting the observations made with PMA and DyeTox13 (Fig. 2).

**TABLE 2:** Cell count of post-pasteurization and post-UV exposure *S*. Typhimurium after CMFDA/PI staining

CMFDA/PI samples detected	Cell count/μL of bacterial suspension			
	CMFDA		PI	
	Mean (cell count/μL)	SD (cell count/μL)	Mean (cell count/μL)	SD (cell count/μL)
Pasteurization (10^5^ CFU/mL)	2.13 × 10^1^	4.25 × 10^0^	1.28 × 10^2^	8.50 × 10^0^
Pasteurization (10^6^ CFU/mL)	1.74 × 10^2^	2.13 × 10^1^	1.12 × 10^3^	3.40 × 10^1^
Pasteurization (10^7^ CFU/mL)	4.29 × 10^2^	2.13 × 10^1^	9.55 × 10^3^	1.72 × 10^3^
UV exposure 10 minutes	4.25 × 10^2^	1.70 × 10^1^	2.13 × 10^1^	4.25 × 10^0^
UV exposure 20 minutes	4.34 × 10^2^	5.10 × 10^1^	5.10 × 10^1^	8.50 × 10^0^
UV exposure 30 minutes	1.11 × 10^2^	2.55 × 10^1^	9.78 × 10^1^	1.28 × 10^1^
Untreated cell (10^6^ CFU/mL)	1.35 × 10^3^	2.89 × 10^2^	2.13 × 10^1^	4.25 × 10^0^
Untreated cell (10^5^ CFU/mL)	3.49 × 10^2^	1.70 × 10^1^	1.28 × 10^1^	4.25 × 10^0^
Untreated cell (10^6^ CFU/mL)	9.14 × 10^2^	3.83 × 10^1^	2.13 × 10^1^	4.25 × 10^0^
Untreated cell (10^7^ CFU/mL)	9.95 × 10^3^	6.38 × 10^2^	7.65 × 10^1^	1.70 × 10^1^

Consistent UV exposure decreased bacterial metabolic activity, as evidenced by reduced cell counts in CMFDA staining with prolonged UV exposure (Fig. S2). Specifically, after 10 minutes of UV exposure, 31.4% of the cells exhibited enzymatic activity, which decreased to 8.2% after 30 minutes. Interestingly, most bacteria remained unstained in the PI row across different UV intensities, suggesting that cell membrane integrity was largely preserved post-UV treatment despite losing cultivability (data not shown). While high-intensity UV exposure slightly increased the proportion of membrane-compromised cells, the differences among treatments were not statistically significant (*P*-value > 0.05).

Therefore, these findings demonstrated the utility of DNA-intercalating dyes such as PMA and DyeTox13 in detecting bacterial viability. Fluorescent microscopy results were consistent with ddPCR findings following the PMA and DyeTox13 treatment. While PMA treatment did not differentiate UV-exposed cells from untreated cells, DyeTox13 and DyeTox13 + EMA successfully distinguished cells subjected to UV exposure. This suggested that DyeTox13-penetrated dead cells with compromised membranes and inhibited PCR implication in cells lacking enzymatic activity.

### Evaluation of the persistence of *Salmonella* cells under different temperatures in flour

To assess *Salmonella*’s persistence and VBNC status in flour, we followed the protocol outlined in Fig. S3. Flour samples inoculated with *S*. Typhimurium were incubated for 3 weeks. Initial *Salmonella* counts were 5, 4, and 3 log10 CFU/gram. These samples, maintaining a water activity of 0.45 ± 0.05, were stored at 4°C, 25°C, and 37°C. To assess the decline of *Salmonella* over time, we employed plate counting, FDA analysis, and viability PCR with these DNA-intercalating dyes.

#### *Salmonella*’s culturability and metabolic activity in the flour using plate counting and FDA analysis

**Fig 4:**
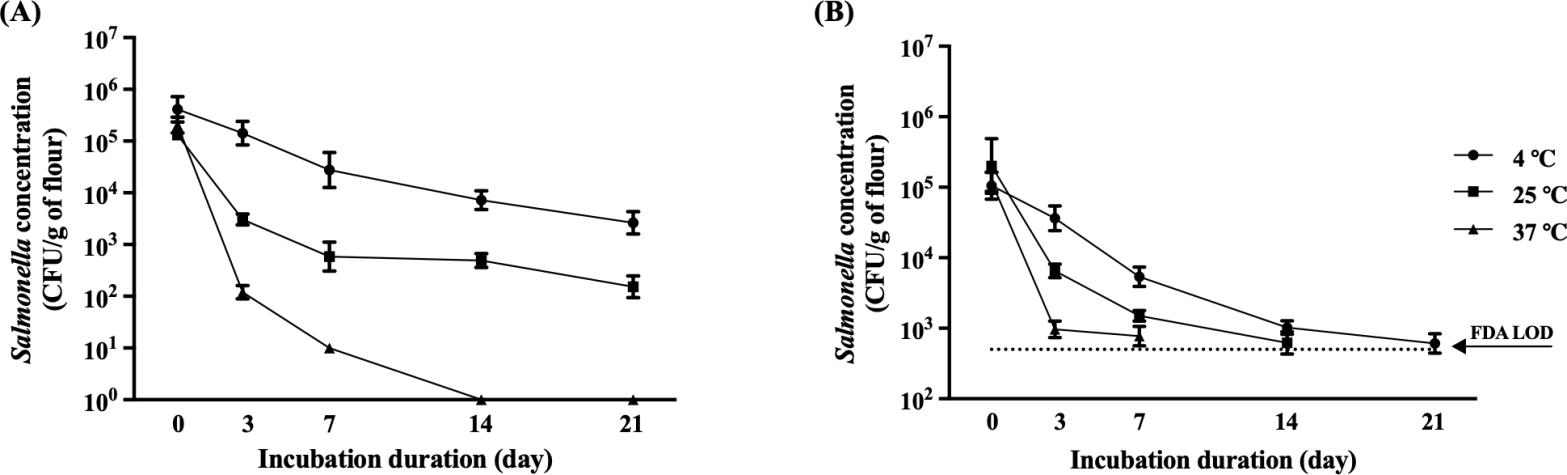
*Salmonella* persistence study with one phase decay regression analysis of detection methods for *Salmonella*-inoculated flour samples. (**A**) Plate counting results of *Salmonella* tested during the incubation period at 4°C, 25°C, and 37°C, respectively. (**B**) FDA staining results were tested by spectrometry at A490 wavelength during the incubation period at 4°C, 25°C, and 37°C, respectively. Based on the FDA standard curve, absorption was converted to CFU per gram of *Salmonella*-inoculated flour samples.

The survival of *Salmonella* cells in flour was monitored over 3 weeks using the plate count method and esterase activity assays. The plate count method indicated that *Salmonella* remained cultivatable for up to 21 days at both 4°C and 25°C but only for 14 days at 37°C, as depicted in Fig. 4A. Regression analysis indicated that the decline in culturable *Salmonella* followed the first-order kinetics (Table 3). The decay rate constants at 4°C, 25°C, and 37°C were calculated as 0.364, 1.297, and 2.490 per day, respectively. The D values (time required for a 1 log reduction) suggested more persistence of *Salmonella* cells at lower temperatures.

**TABLE 3:** Comparison of one phase decay regression analysis of plate counting and FDA assays for *Salmonella*-inoculated flour during the incubation period^*a*^^,^^*b*^^,^^*c*^

Storage condition (Temp.)	Nonlinear regression (y = (y_0_ -Plateau) *exp(-k*x) +Plateau)							
	Culturable cells (Plate counting method)				Enzymatically active cells (FDA analysis)			
	k	Plateau	D value (day)	R^2^	k	plateau	D value (day)	R^2^
4°C	0.35	0	6.58	0.99	0.36	0	6.40	0.99
25°C	1.25	0	1.84	0.92	1.17	0	1.97	0.99
37°C	2.48	0	0.93	0.99	1.71	0	1.35	1

^*a*^ k (decay rate): k represents the rate constant of the decay process.

^*b*^ D value: D value refers to the time required for a one log reduction from its initial value.

^*c*^ Plateau: Plateau represents the final stable value that the *Salmonella* concentration tended to reach after the incubation process.

FDA was used to evaluate the viability of metabolically active cells in flour. FDA, capable of penetrating viable bacterial membranes, is rapidly hydrolyzed by enzymes such as esterase, lipases, and proteases, serving as an indicator of cell viability. As shown in Fig. 4B, flour samples inoculated with *Salmonella* and stored at 4°C showed a gradual decline in metabolic activities over time, decreasing from 3 × 10^5^ to 8 × 10^2^ CFU/g of flour. Samples stored at 25°C and 37°C exhibited a significant reduction within the first 3 days of incubation. Metabolically active cells became nearly undetectable after seven days at 25°C and 14 days at 37°C. Notably, the D values from the plate counting method and FDA analysis at 4°C were 6.58 and 6.40 days (Table 3), implying that metabolic activity and culturability were similar due to reduced metabolic rate and energy conservation. However, at 37°C, the D values of the FDA were slightly longer than that determined by the plate count method, suggesting the presence of metabolically active cells in a non-culturable state.

#### Survival of *Salmonella* cells measured by viability PCR

In this study, we utilized DNA-intercalating dyes, specifically PMA, DyeTox13, and DyeTox13 + EMA, to evaluate the viability of *Salmonella* cells in flour. Each DNA-intercalating dye was selected for its specificity in indicating distinct cellular states: untreated samples representing all DNA (live and dead cells), PMA for cells with intact membranes, DyeTox13 for cells exhibiting both intra- and extra-cellular enzymatic activities, and DyeTox13 +EMA for metabolically active cells within the intracellular environment.

**Fig 5:**
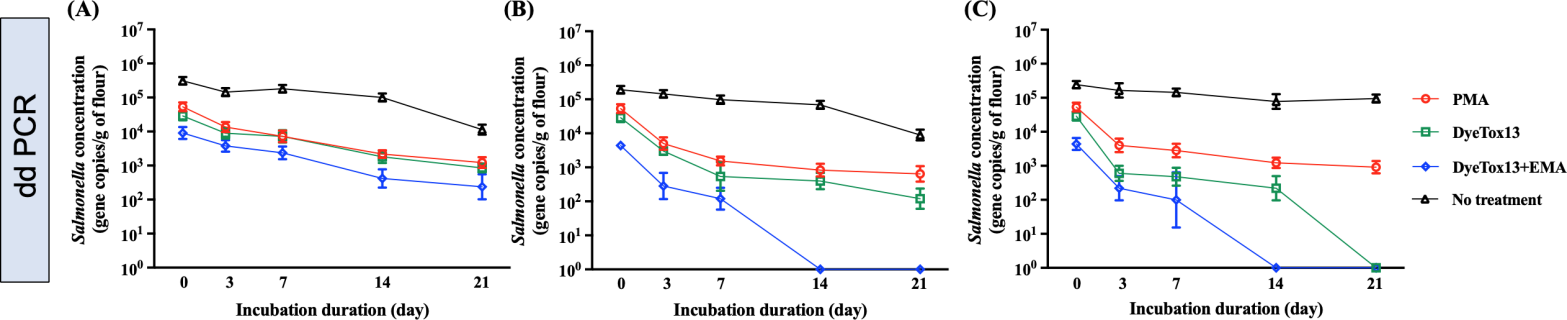
DNA-intercalating dye-exposure viability tests, including PMA, DyeTox13, DyeTox13 + EMA, and no treatment results, respectively, for testing the *Salmonella*’s physiological status during the incubation period at different storage temperatures. (**A**), (**B**), and (**C**) referred to the flour samples incubated at 4°C, 25°C, and 37°C, respectively.

We monitored the persistence of *Salmonella* at various temperatures over 3 weeks, quantifying the *invA* gene in flour using ddPCR, as shown in Fig. 5. Our results indicated that *Salmonella*’s survival in flour was temperature dependent, with notably prolonged persistence at the lower temperature of 4°C. This trend was consistent across all categories: total DNA, membrane-intact cells, enzymatically active cells, and metabolically active cells (Fig. 5A). Interestingly, the decay rates and D values of DNA in untreated samples remained consistent across temperatures, with decay rates of total DNA at 0.09, 009, and 0.08 day^−1^ at 4, 25, and 37°C in Table 4, respectively. The survival rates of *Salmonella* cells treated with PMA, DyeTox13, and DyeTox13 + EMA at 4°C were similar, ranging from 0.40 and 0.42 day^−1^ (Table 4), suggesting a stable physiological status of *Salmonella* cells in flour at this low temperature. However, there are distinct differences in the decay rates of PMA and other DNA intercalating dyes, such as DyeTox13 and DyeTox13 + EMA. Specifically, the D values of DyeTox13 + EMA at 25 and 37°C were 1.93 and 1.84 days, respectively, compared to 2.95 and 2.37 days for PMA, indicating a rapid loss of metabolic activity at higher temperatures (Table 4).

**TABLE 4:** Comparison of one phase decay regression analysis of dye-ddPCR assays for *Salmonella*-inoculated flour during the incubation period^*a*^^,^^*b*^^,^^*c*^

Storage condition (Temp.)	Nonlinear regression (y = (y_0_ -Plateau) x e^(−k*x)^ +Plateau)															
	No treatment				PMA				DyeTox13				DyeTox13 + EMA			
	k	Plateau	D value (day)	R^2^	k	Plateau	D value (day)	R^2^	k	Plateau	D value (day)	R^2^	k	Plateau	D value (day)	R^2^
4°C	0.09	4000	25.6	0.83	0.40	1578	5.76	0.96	0.41	2346	5.62	0.97	0.42	953	5.48	0.96
25°C	0.09	4000	25.6	0.96	0.78	952	2.95	0.99	0.81	363	2.84	0.99	1.19	62	1.93	0.99
37°C	0.08	6000	28.8	0.86	0.96	1493	2.37	0.99	1.17	111	1.97	0.99	1.25	53	1.84	0.99

^*a*^ k (decay rate): k represents the rate constant of the decay process.

^*b*^ D value: D value refers to the time required for a one log reduction from its initial value.

^*c*^ Plateau: Plateau represents the final stable value that the *Salmonella* concentration tended to reach after the incubation process.

**Fig 6:**
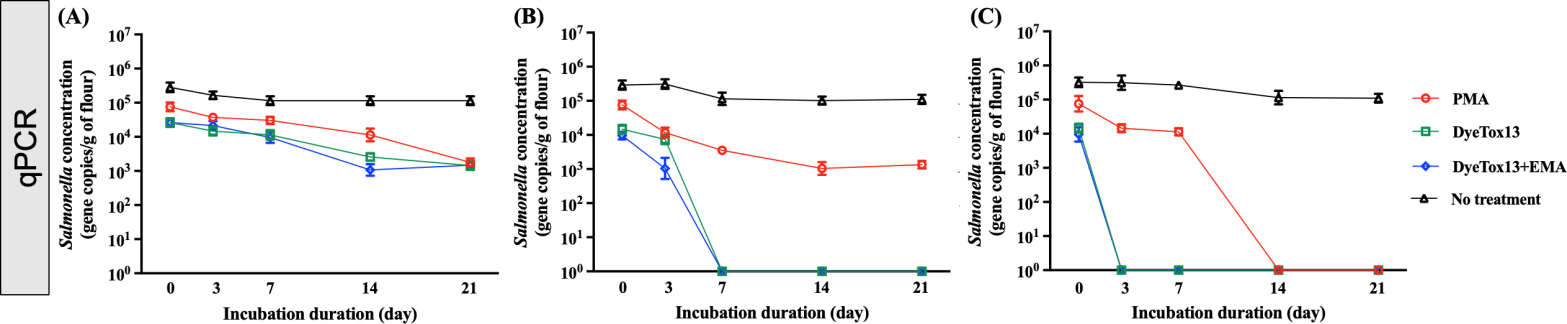
DNA-intercalating dye-exposure viability tests, including PMA, DyeTox13, DyeTox13 + EMA, and no treatment results, respectively, for testing the *Salmonella*’s physiological status during the incubation period at different storage temperatures. (**A**), (**B**), and (**C**) referred to the flour samples incubated at 4°C, 25°C, and 37°C, respectively.

In addition, we assessed the persistence of viable *Salmonella* cells using qPCR with DNA-intercalating dyes. Consistent with ddPCR findings, temperature variance did not significantly influence the decay rates of untreated samples. At 4°C, the decay rates of all DNA-intercalating dyes were comparable, ranging between 0.15 and 0.16 day^−1^. However, as depicted in Fig. 6B and C, both DyeTox13 and DyeTox13 + EMA rendered the *invA* gene undetectable after 7 and 3 days at 25°C and 37°C, respectively. In contrast to ddPCR, qPCR exhibited lower sensitivity in detecting the flour target gene at these temperatures. Notably, the samples treated with DyeTox13 and DyeTox13 + EMA at 37°C showed decay rates markedly different from those treated with PMA, which exhibited a 0.56 day^−1^ (Table 5).

**TABLE 5:** Comparison of one phase decay regression analysis of dye-qPCR assays for *Salmonella*-inoculated flour during the incubation period^*a*^^,^^*b*^^,^^*c*^^,^^*d*^

Storage condition (Temp.)	Nonlinear regression (y = (y_0_ -Plateau) x e^(−k*x)^ + Plateau)															
	No treatment				PMA				DyeTox13				DyeTox13 + EMA			
	k	Plateau	D value (day)	R^2^	k	Plateau	D value (day)	R^2^	k	Plateau	D value (day)	R^2^	k	Plateau	D value (day)	R^2^
4°C	0.06	5000	38.3	0.68	0.16	3180	14.39	0.96	0.15	35	15.35	0.97	0.15	0	15.35	0.95
25°C	0.07	5000	32.9	0.75	0.35	0	6.40	0.95	0.45	533	5.12	0.93	0.74.	0	3.11	0.99
37°C	0.06	5000	38.3	0.90	0.56	2836	4.11	0.98	N.D.	N.D.	N.D.	N.D.	N.D.	N.D.	N.D.	N.D.

^*a*^ k (decay rate): k represents the rate constant of the decay process.

^*b*^ D value: D value refers to the time required for a one log reduction from its initial value.

^*c*^ Plateau: Plateau represents the final stable value that the *Salmonella* concentration tended to reach after the incubation process.

^*d*^ N.D. refers to not detectable.

Our comparative analysis between ddPCR and qPCR in evaluating bacterial viability under varying storage conditions revealed that qPCR was less effective in accurately determining the rate constant and D values, especially at 25 and 37°C. By contrast, ddPCR demonstrated robustness in providing these critical data points. Notably, ddPCR results revealed a rapid loss of metabolic activity, even though the membrane remained intact across varying storage temperatures. Moreover, treatments with DyeTox13 and DyeTox13 + EMA successfully differentiated between enzymatically active cells and those with intact or compromised membranes. This finding highlights ddPCR’s enhanced sensitivity and precision in accurately tracking the temporal dynamics of bacterial viability, a crucial aspect of food safety.

### The correlation between the plate counting method and viability PCR in flour samples across varying temperatures

**Fig 7:**
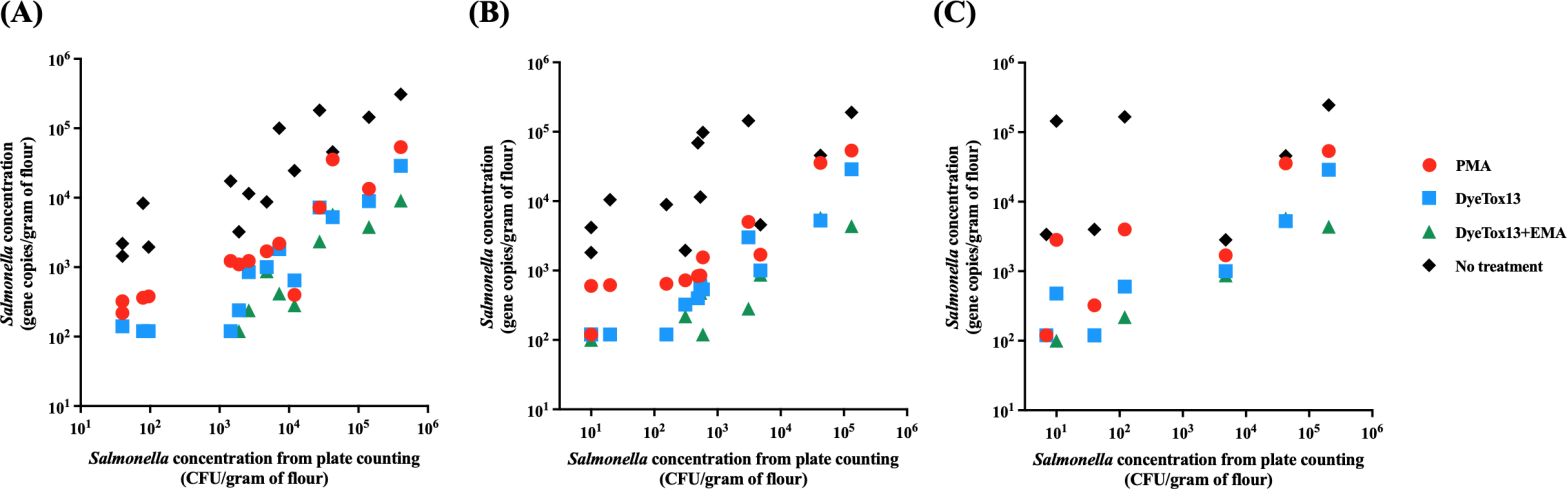
Comparison between plate counting and DNA-intercalating dye-viability test including PMA, DyeTox13, DyeTox13 + EMA, and no treatment results, respectively. To test the *Salmonella*’s physiological status during incubation at different storage temperatures under different doses of *Salmonella* concentration. (**A**), (**B**), and (**C**) referred to the flour samples incubated at 4°C, 25°C, and 37°C and tested by ddPCR, respectively.

In this study, we conducted a correlation analysis to compare the traditional plate method with the applications of DNA-intercalating dyes in ddPCR across various temperatures. We analyzed the results at three different cell concentrations (3-log, 4-log, and 5-log) to assess the effectiveness of DNA-intercalating dyes in estimating the viable cell count as a reliable alternative for rapid detection of bacterial viability. The analysis demonstrated a significant correlation between gene concentrations in samples treated with DNA-intercalating dyes and untreated samples and the cell counts obtained from the plate count method at 4°C (*P*-value < 0.05 and R^2^ >0.78). However, at 25 and 37°C, the correlation between untreated samples and the plate counting method did not show a statistically significant relationship (*P*-value > 0.05 or R^2^ <0.5). A strong relationship was observed between the DyeTox13 dyes (DyeTox13 and DyeTox13 + EMA) and the plate counting method, suggesting that DyeTox13 could serve as an alternative method for the rapid detection of *Salmonella* cells in flour (Fig. 7). Notably, at 37°C, both DyeTo13 and DyeTox13 + EMA treatments demonstrated a strong correlation with the plate counts (*P*-value < 0.003 or R^2^ >0.85), indicating high predictability or reliability in the correlation analysis (Table 6).

**TABLE 6:** Comparison between plate counting and dye-viability ddPCR assays during the incubation period at different storage temperatures under different doses of *Salmonella* concentration

Dye treatment type	4°C			25°C			37°C		
	*P* < 0.05	*P* value (two-tailed)	R^2^	*P* < 0.05	*P* value (two-tailed)	R^2^	*P* < 0.05	*P* value (two-tailed)	R^2^
PMA	****	<0.0001	0.7828	****	<0.0001	0.8403	*	0.0204	0.6917
DyeTox13	****	<0.0001	0.8524	****	<0.0001	0.8954	**	0.0031	0.8501
DyeTox13 + EMA	***	<0.0001	0.8545	**	0.0027	0.7998	***	0.0007	0.9568
No treatment	****	<0.0001	0.8013	*	0.0204	0.4309	N.S.^*a*^	0.5115	0.09075

^*a*^ N.S. refers to no significant difference.

## DISCUSSION

Our study demonstrated significant advancements in detecting the *invA* gene of *Salmonella*, employing ddPCR in conjunction with DNA-intercalating dyes such as PMA, DyeTox13, and DyeTox13 + EMA. This approach not only assesses the viability of *Salmonella* cells but also facilitates a comprehensive comparison with conducting traditional qPCR and plate counting methods. Expanding on ddPCR’s proven ability to detect *Salmonella*, our research extends its utility to quantifying viable *Salmonella*, particularly in evaluating inactivation effectiveness and persistence in wheat flour. Also, we demonstrated that ddPCR approaches with DNA-intercalating dyes were highly sensitive and precise in detecting the *invA* gene of *S*. Typhimurium, excelling in quantifying targets of low abundance. The combination of ddPCR with DNA-intercalating dyes overcame the limitations of traditional culture-based methods and offered greater sensitivity than the qPCR (25, 37). Thus. The use of ddPCR with DNA-intercalating dyes provides new insights into the viability states of *Salmonella* following inactivation and its persistence in flour under varying storage conditions, contributing valuable information to the understanding of *Salmonella* contamination dynamics in food processing and storage for food safety and public health.

The use of ddPCR for the detection of the *invA* gene of *S*. Typhimurium showed significant improvement in sensitivity in identifying low-abundance targets over traditional qPCR methods, particularly in terms of the LOD and LOQ compared to qPCR. This increased sensitivity is attributed to ddPCR’s digital partitioning of reactions and its capacity for absolute quantification. Unlike qPCR, which quantifies targets based on real-time amplification and fluorescent signals during the exponential phase, ddPCR estimates target concentration by analyzing end-point fluorescence signals across multiple partitions, converting the qPCR’s continuous signal into a binary format. This method eliminates the need for calibration curves, reducing variability from fluctuating reaction efficiencies (38, 39). ddPCR’s accuracy and performance are governed by binomial statistics, and its ability to distribute target molecules across numerous partitions minimizes competition effects, enhancing sensitivity. By contrast, qPCR’s reliance on relative quantification and primer amplification efficiency can reduce sensitivity, especially at low concentrations (40). The higher LOQ in qPCR indicated a narrower dynamic range compared to ddPCR. By contrast, ddPCR’s broader range makes it particularly suitable for detecting rare sequences in low-level pathogen detection applications.

In this study, we evaluated the effectiveness of DNA-intercalating dyes, such as PMA, DyeTox13, and DyeTox13 + EMA, in combination with ddPCR and qPCR techniques to distinguish viable from non-viable *S*. Typhimurium cells post-pasteurization and UV exposure. Our results showed marked differences in gDNA concentrations between samples treated with these dyes and untreated controls, demonstrating the utility of viability PCR in discriminating between viable and dead cells. Post-pasteurization, while plate count assays showed no culturable *S*. Typhimurium cells, both ddPCR and qPCR with DNA-intercalating dyes detected the presence of the bacteria across all samples ranging from 10^1^ to 10^2^ gene copies/μL, suggesting the survival of a small bacterial population post-pasteurization. This highlights the potential of using membrane integrity as a rapid post-pasteurization viability assessment tool. Furthermore, ddPCR and qPCR revealed that some *S*. Typhimurium cells might enter a VBNC state after pasteurization, maintaining membrane integrity and enzymatic activity (41). The differential responses of *S*. Typhimurium to varying UV dosages were also notable. The combination of EMA with DyeTox13 proved particularly effective in assessing the metabolic activity of UV-exposed cells. ddPCR analysis using these dyes indicated that UV radiation primarily affected cellular metabolism, with most *S*. Typhimurium cells showing halted metabolic activities but intact membranes (8, 34). CMFDA and PI staining, used alongside fluorescence microscopy, corroborated the ddPCR findings. Notably, DyeTox13 and DyeTox13 + EMA effectively differentiated cells post-UV exposure penetrated dead cells with compromised membranes, and inhibited PCR amplification in cells lacking enzymatic activity.

Our assessment of *Salmonella* survival in flour over 3 weeks revealed temperature-dependent viability. The plate count method showed that *Salmonella* remained cultivable for extended periods at lower temperatures, up to 21 days at 4°C and 25°C, in contrast to just 14 days at 37°C. Complementing these results, using the FDA to assess metabolically active cells indicated a notable reduction in metabolic activity at higher temperatures. This aligned with findings that foodborne pathogens like *Salmonella* and *E. coli* O121, while detectable in wheat flour, had their survivability markedly influenced by storage temperature (42). Notably, at 37°C, *Salmonella* populations declined more rapidly than at room temperature (3), indicating that cooler temperatures may promote *Salmonella*’s longevity in flour. This phenomenon could be explained by various mechanisms: a decrease in membrane fluidity, which affects cellular transport and signaling; stabilization of RNA structures, impacting gene expression; and diminished efficiency of critical cellular processes, including transcription, translation, and degradation (43, 44). Furthermore, lower temperatures likely suppress the activity of critical enzymes and biochemical pathways that are more active at higher temperatures (45).

Using DNA-intercalating dyes (PMA, DyeTox13, DyeTox13 + EMA), our study provided insights into *Salmonella* cell viability in low-water activity environments, such as flour. We observed a notable extension in *Salmonella* persistence at 4°C. Despite varying temperatures, the decay rates of DNA in dye-treated samples were consistent, indicating maintained cellular integrity and reduced metabolic activity in *Salmonella* at lower temperatures. Conversely, the rapid decline in metabolic activity at higher temperatures, particularly in samples treated with DyeTox13 and DyeTox13 + EMA, paralleled the survival trends of culturable *Salmonella* at 25 and 37°C. This highlighted the effectiveness of these dyes in identifying viable cells, demonstrating their potential for food safety applications. Comparing the survival of culturable *Salmonella* (Fig. 4) with our findings, it appeared that storage temperatures might induce a transition to VBNC states in *Salmonella*. For example, D values in samples treated with DyeTox13 and DyeTox13 + EMA at 25°C and 37°C exceeded those from culturable *Salmonella*, suggesting they maintained metabolic activity. However, lower D values in dye-treated samples at reduced temperatures implied prevention of entry into the VBNC state. Previous studies indicated that environmental conditions such as acidic pH, low temperature, and nutrient deficiency could inhibit the transition to the VBNC state (41, 46).

Our correlation analysis between the traditional plate counting method and viability PCR using DNA-intercalating dyes revealed significant relationships at different temperatures (Fig. 7). Integrating viability PCR for *Salmonella* quantification could complement existing methods, addressing challenges in detecting foodborne pathogens. Recently, the successful application of PMA with qPCR for quantifying viable cells of pathogens like *Salmonella* and *E. coli* O157:H7 supported this approach (21, 47–51). In our study, the correlation was particularly strong with the DyeTox13 dyes, suggesting their effectiveness as rapid detection methods for *Salmonella* in flour. Notably, at higher temperatures (37°C), both DyeTox13 and DyeTox13 + EMA treatments strongly correlated with plate counts, indicating their reliability in detecting *Salmonella* viability. This study was the first to apply DNA-intercalating dyes with ddPCR to quantify viable *Salmonella* in flour. The ddPCR-based method proposed its rapidity, selectivity, and specificity, bridged a crucial gap in traditional molecular assays, which often failed to detect *Salmonella* cells during food processing or within low-moisture food products like flour (40, 52). Moreover, our approach not only monitors the physiological status of *Salmonella* in food but also detects inactivation-injured cells that conventional cultivation methods might miss (53). Our study introduced a novel approach for detecting and characterizing the physiological state of *Salmonella*, utilizing the *Salmonella enterica* Serova Typhimurium Strain as a model organism to investigate their survival in a flour environment. We, however, recognize the limitation of focusing solely on this strain. Hence, we acknowledged the necessity of including various *Salmonella* strains in future research. This might be particularly critical for strains associated with flour-related outbreaks, such as *Salmonella* Infantis, which might possess genetic modifications enhancing their survival in flour. In addition, the survival of *Salmonella* strains under different storage conditions, including moisture content and temperature, can differ because certain strains might survive better in dry conditions (e.g., flour). Variations in the ability to enter a VBNC state under adverse conditions can also influence the persistence of different *Salmonella* strains in food products during storage. Understanding how different strains adapt and survive under these conditions can inform better control strategies and improve food safety standards.

Environmental stresses could induce bacterial pathogens to enter a VBNC state, often eluding detection by standard laboratory techniques. This poses a significant challenge for the food industry, mainly due to the potential resuscitation of these pathogens under favorable conditions (54). In this study, we employed DNA-intercalating dyes, such as PMA, DyeTox13, and DyeTox13 + EMA, to differentiate enzymatically active cells, those with intact membranes, VBNC, and dead cells. As measured by plate counting techniques and DNA-intercalating dyes, the observed decay rates and D values suggested the possible presence of VBNC pathogens, posing a serious risk to food safety and public health. Previous research highlighted the importance of VBNC pathogens in public health and food safety, linking them to foodborne outbreaks, albeit without direct evidence of causation (4, 55). This absence of direct evidence is likely due to the challenges in detecting VBNC cells, as many illnesses are attributed to unspecified or unidentified agents, potentially overlooking VBNC pathogens (56). Therefore, the combination of ddPCR and DNA intercalating dyes could offer valuable insights into the presence and dynamics of VBNC *Salmonella* in food. Food is often subjected to a complex range of environmental conditions, where factors like pH, water activity, disinfectants, chemical composition, high-pressure CO_2_, varying temperatures, storage duration, decontamination treatments, pasteurization, and modified atmosphere packaging can induce bacteria into a VBNC state. Our study revealed that VBNC cells can evade detection by conventional plate counting methods and withstand inactivation processes, including pasteurization and UV treatment. Consequently, ddPCR combined with DNA-intercalating dyes emerges as a promising alternative method for the rapid, sensitive, cost-effective, and user-friendly detection of foodborne pathogens and their VBNC states, thereby mitigating the risk of foodborne diseases.

## SUPPLEMENTAL MATERIAL

Supplemental figures spectrum.00249-24-s0001.pdf Fig. S1 to S3.

Supplemental tables spectrum.00249-24-s0002.DOCX Tables S1 and S2.

ASM does not own the copyrights to Supplemental Material that may be linked to, or accessed through, an article. The authors have granted ASM a non-exclusive, world-wide license to publish the Supplemental Material files. Please contact the corresponding author directly for reuse.
