# Supplementary material for: Quantitative detection and survival analysis of VBNC Salmonella Typhimurium in flour using droplet digital PCR and DNA-intercalating dyes
Source: Microbiol Spectr. 2024 Jul 8;12(8):e00249-24. doi: 10.1128/spectrum.00249-24 (PMC11302299; doi:10.1128/spectrum.00249-24)
Supplement: Supplemental figures — Fig. S1 to S3. [file spectrum.00249-24-s0001.pdf]

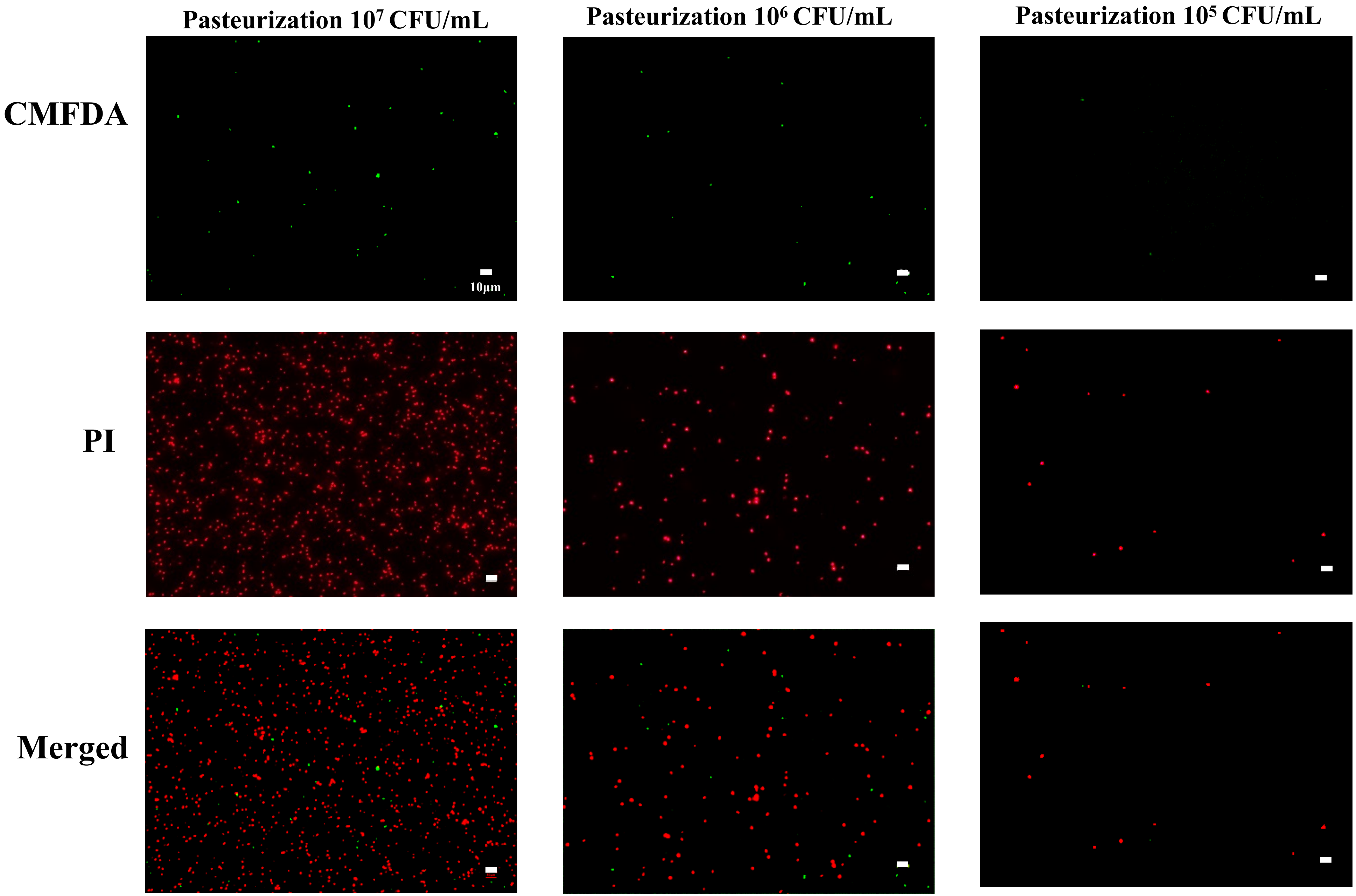

Figure S1. Enzymatic-active/dead staining of post-pasteurization *S.* Typhimurium cells under a fluorescence microscope. Enzymatic-active cells (CMFDA-stained) appear green, while membrane-compromised dead bacteria are shown in red (PI-stained) (scale bar=10 μm).

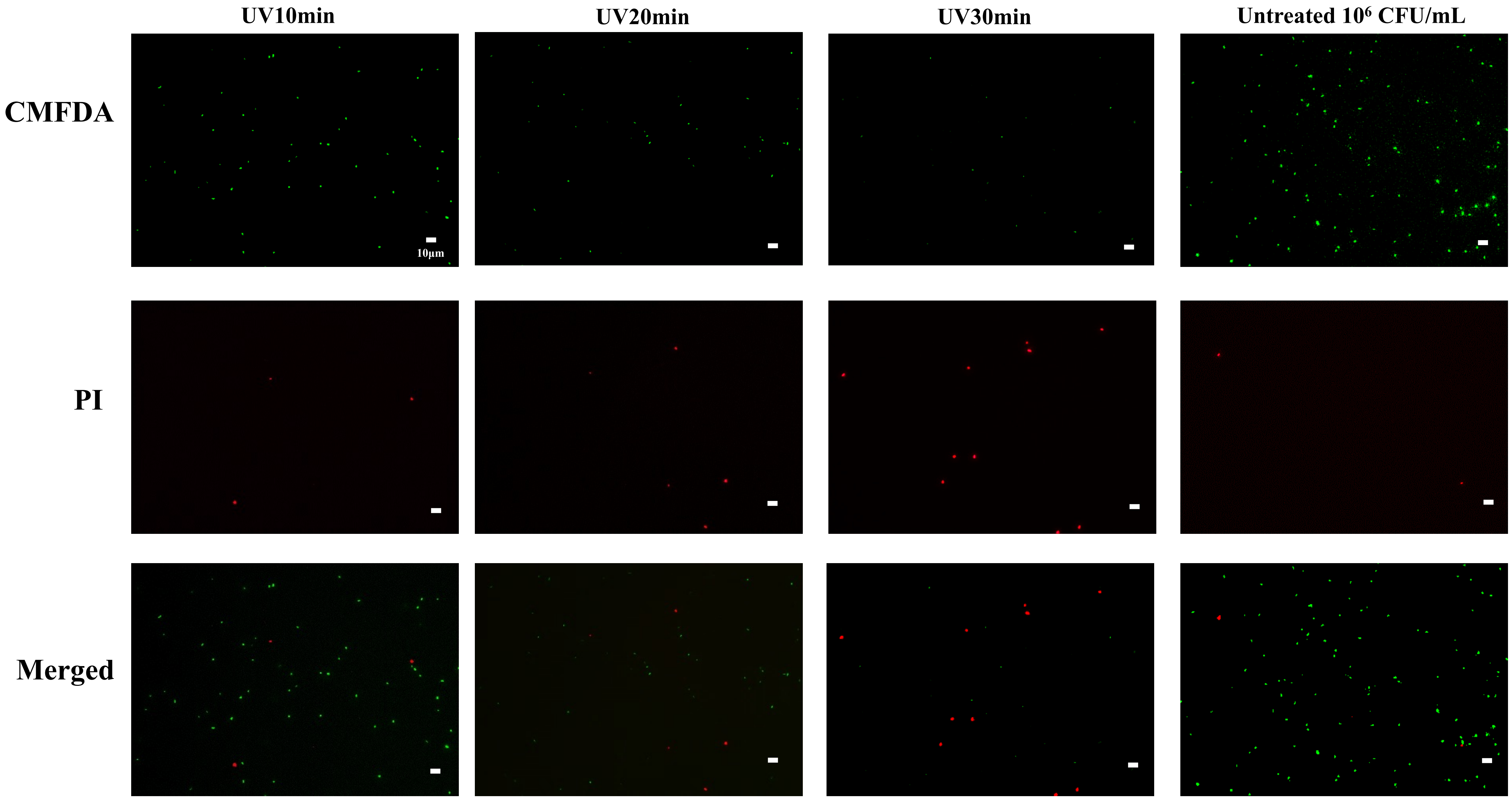

Figure S2. Enzymatic-active/dead staining of post-UV exposure *S*. Typhimurium cells under a fluorescence microscope. Enzymatic-active cells (CMFDA-stained) appear green, while membrane-compromised dead bacteria are shown in red (PI-stained) (scale bar=10 μm).

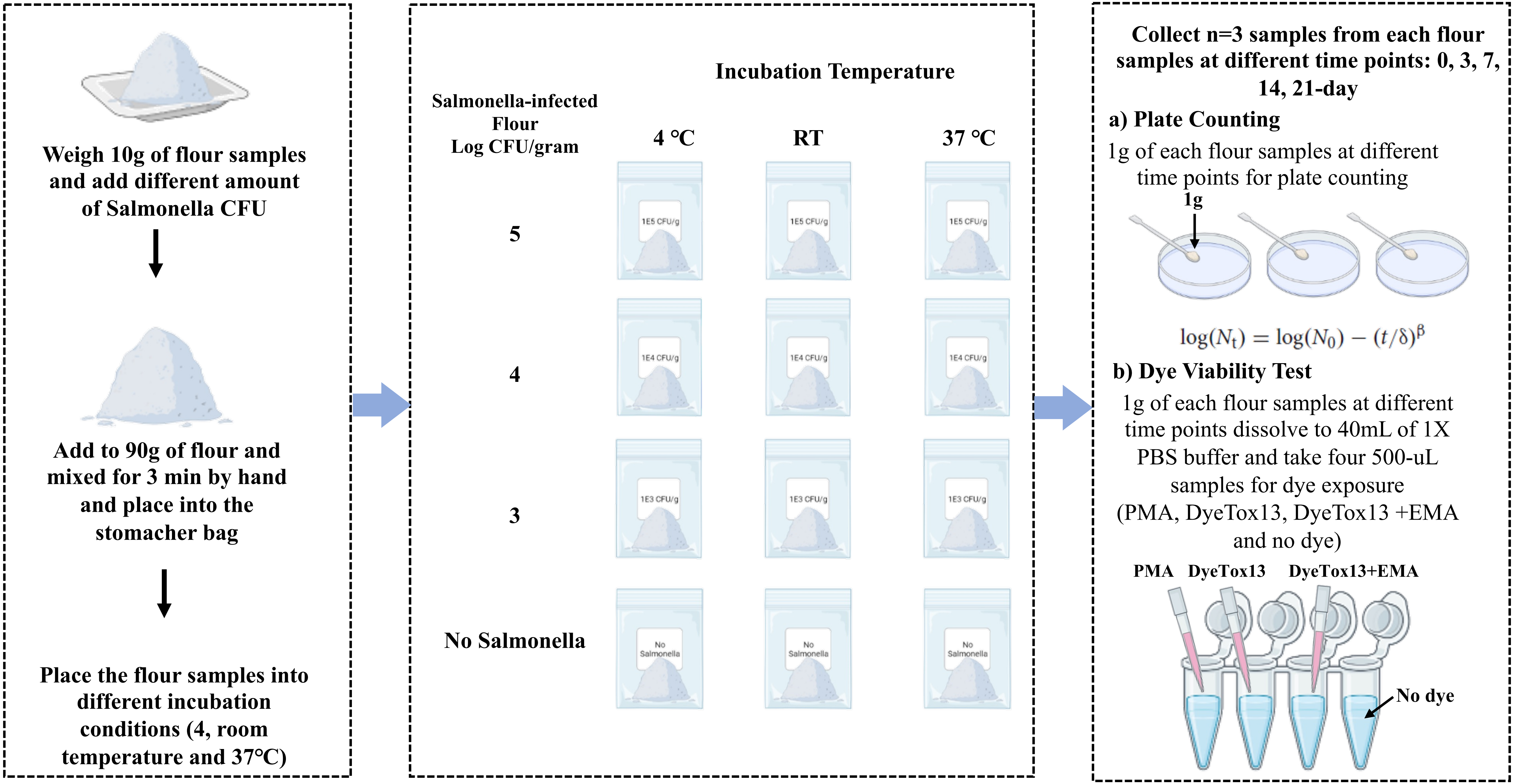

Figure S3. Schematic representation of the *Salmonella* persistence experiment in flour conducted at various incubation temperatures.
