# Supplementary material for: Quantitative detection and survival analysis of VBNC Salmonella Typhimurium in flour using droplet digital PCR and DNA-intercalating dyes
Source: Microbiol Spectr. 2024 Jul 8;12(8):e00249-24. doi: 10.1128/spectrum.00249-24 (PMC11302299; doi:10.1128/spectrum.00249-24)
Supplement: Supplemental tables — Tables S1 and S2. [file spectrum.00249-24-s0002.docx]

**Table S1.** Quantification of viable cells using PMA-, DyeTox13-, DyeTox13+EMA-dye treatment, and no dye treatment after pasteurization

| **gDNA Samples Detected** | | **ddPCR** | **qPCR** |
| --- | --- | --- | --- |
|  |  | **Mean ± SD**  **(gene copies/μL)** | **Mean ± SD**  **(gene copies/μL)** |
| **Pasteurization**  **(10^5^ CFU/mL)** | **PMA** | **2.20 ± 0.8 ×10^1^** | **4.08 ± 0.3 ×10^1^** |
|  | **DyeTox13** | **1.18 ± 0.5 ×10^1^** | **3.04 ± 0.6 ×10^1^** |
|  | **DyeTox13+EMA** | **3.60 ± 0.09 ×10^1^** | **9.70 ± 0.2 ×10^1^** |
|  | **Untreated** | **1.60 ± 0.06 ×10^3^** | **6.60 ± 0.7 ×10^3^** |
| **Pasteurization**  **(10^6^ CFU/mL)** | **PMA** | **1.08 ± 0.2 ×10^2^** | **2.92 ± 0.1 ×10^2^** |
|  | **DyeTox13** | **2.20 ± 0.07 ×10^1^** | **7.68 ± 3.2 ×10^1^** |
|  | **DyeTox13+EMA** | **3.80 ± 0.3 ×10^0^** | **1.00 ± 0.2 ×10^1^** |
|  | **Untreated** | **2.24 ± 0.03 ×10^4^** | **8.64 ± 2.6 ×10^4^** |
| **Pasteurization**  **(10^7^ CFU/mL)** | **PMA** | **7.60 ± 1.5 ×10^1^** | **6.49 ± 0.9×10^2^** |
|  | **DyeTox13** | **5.20 ± 1.2 ×10^1^** | **1.40 ± 0.1 ×10^3^** |
|  | **DyeTox13+EMA** | **2.00 ± 0.7 ×10^1^** | **1.11 ± 0.1×10^2^** |
|  | **Untreated** | **3.41 ± 0.02 ×10^4^** | **9.87 ± 3.2 ×10^5^** |

**Table S2.** Quantification of viable cells using PMA-, DyeTox13-, DyeTox13+EMA-dye treatment, and no dye treatment after UV exposure

| **gDNA Samples Detected** | | **ddPCR** | **qPCR** |
| --- | --- | --- | --- |
|  |  | **Mean ± SD**  **(gene copies/μL)** | **Mean ± SD**  **(gene copies/μL)** |
| **UV-10min** | **PMA** | **1.1 ± 0.1 ×10^4^** | **1.3 ± 0.3 ×10^4^** |
|  | **DyeTox13** | **2.2 ± 0.2 ×10^2^** | **3.1 ± 0.7 ×10^1^** |
|  | **DyeTox13+EMA** | **2.6 ± 0.8 ×10^1^** | **N.D.** |
|  | **Untreated** | **2.7 ± 0.01 ×10^4^** | **2.5 ± 0.7 ×10^4^** |
| **UV-20min** | **PMA** | **9.3 ± 0.2 ×10^3^** | **1.6 ± 0.02×10^4^** |
|  | **DyeTox13** | **5.6 ± 1.3 ×10^1^** | **2.4 ± 0.7 ×10^1^** |
|  | **DyeTox13+EMA** | **1.2 ± 0.8 ×10^1^** | **N.D.** |
|  | **Untreated** | **4.1 ± 0.01 ×10^4^** | **1.8 ± 0.4 ×10^4^** |
| **UV-30min** | **PMA** | **8.9 ± 0.2 ×10^3^** | **7.7 ± 0.2 ×10^3^** |
|  | **DyeTox13** | **3.4 ± 0.6 ×10^2^** | **5.0 ± 1.7 ×10^1^** |
|  | **DyeTox13+EMA** | **6.4 ± 4.5 ×10^0^** | **N.D.** |
|  | **Untreated** | **7.0 ± 0.1 ×10^4^** | **3.2 ± 0.2 ×10^4^** |

N.D. refers to not detectable (below limit of detection).
